# Viral Impact on Prokaryotic and Microalgal Activities in the Microphytobenthic Biofilm of an Intertidal Mudflat (French Atlantic Coast)

**DOI:** 10.3389/fmicb.2015.01214

**Published:** 2015-11-10

**Authors:** Hélène Montanié, Margot G. De Crignis, Johann Lavaud

**Affiliations:** UMRi 7266 ULR- Centre National de la Recherche Scientifique, LIENSs, Institut du Littoral et de l'Environnement, Université de La RochelleLa Rochelle, France

**Keywords:** virus, prokaryotes, microphytobenthos, photosynthesis, sediment, mudflat

## Abstract

This is the first report on viriobenthos activity within the microbial biofilm located at the top-surface of the intertidal mudflat during emersion in Marennes-Oléron Bay (France). By combining *in situ* and *ex situ* approaches, the viral production (VP) was linked to the dynamics of prokaryotes and microphytobenthos (MPB). VP averaged 2–4 × 10^8^ viruses ml^−1^ h^−1^. VP correlated positively with the Virus to Prokaryote Ratio, and both were correlated negatively with the water content. The virus-induced mortality of prokaryotes was lower in winter than in summer (6.8 vs. 39.7% of the production) and the C-shunting may supply 2–12% of their Carbon Demand, respectively. VP accounted for 79% of loss in Prokaryotes but the response was delayed compared to the increase in VP suggesting a simultaneous release of viruses of MPB origin. This hypothesis is supported by capsid-sizing of virions by transmission electronic microscopy and bioassays. Harvesting and *ex situ* maintenance of top-surface sediments was carried out to monitor the dynamics of viruses, prokaryotes and MPB after inoculation with benthic or planktonic viruses. Benthic viruses modified the prokaryotic and MPB dynamics and decreased the photosynthesis efficiency in contrast to planktonic viruses that impacted MPB but not the prokaryotes.

## Introduction

Microbial communities are structured by the intrinsic activities of viruses (Sime-Ngando, 2014) in terms of diversity and dynamics, directly through the process of virus-mediated cell lysis and changes in metabolic properties and/or indirectly by the bioavailability of significant amounts of viral lysates that may infer a reduction in competition pressure (Suttle, 2007). The viral shunt of matter (Wilhelm and Suttle, 1999) tends thus (i) to promote the recycling of carbon and nutrients by bacterial remineralizers (Suttle, 2007), (ii) to reduce the transfer of organic carbon to higher trophic levels (Fuhrman, 1999) and (iii) overall to lubricate the microbial food-web on a short-time scale (Weinbauer et al., 2009). In the water column, up to 25% of the bacterial community (Weinbauer, 2004) may be infected and viruses are assumed to account for 20–40% of the daily mortality of the standing stock of planktonic bacteria (Suttle, 2007) and for 10–30% of the daily loss of bacterial production (Fuhrman, 1999). They affect both the biomass of phytoplankton (i.e., 10–50% of microalgae, Gastrich et al., 2004) and the photosynthesis, probably through metabolic reprogramming (Hurwitz et al., 2013), and reduce their primary productivity (Suttle et al., 1990; Juneau et al., 2003). Concerning the benthic deep-sea body, virus-induced mortality could globally represent as high as 80% of the benthic prokaryotic heterotrophic production (Danovaro et al., 2008a).

The so-called phage kills the winner concept (KtW; Thingstad and Lignell, 1997) was tested on a panel of planktonic and benthic data sets (Winter et al., 2010) and revealed a paradox in freshwater benthos where there is an apparent low infectivity of viruses together with an high abundance of bacterial hosts and viruses (Filippini et al., 2006). However, to question the universality of this paradox, the panel of environments considered needs to be enlarged, particularly in light of the fact that information on viriobenthos is scarce. Although, analyses have been performed on viriobenthos from a variety of sediments (rewiewed by Danovaro et al., 2008b; Middelboe et al., 2011; Helton et al., 2012) including surface layers in subtidal estuaries, coastal areas, continental lakes, and deep-ocean sediments, there is only parceled information of abundance concerning viriobenthos in the sediments of intertidal mudflats (Montanié et al., 2014; Careira et al., 2015).

In Western European macrotidal estuaries and semi-enclosed bays, the primary productivity of intertidal mudflats is supported by motile microalgae (microphytobenthos, MPB) which are generally dominated by diatoms and form the main component of a dense biofilm at the surface of the sediment at low tide (Pierre et al., 2012). The MPB biofilm is stabilized by the exudation of Exocellular Polymers Substances (EPS) by both microalgae and prokaryotes (Orvain et al., 2014a). These epipelic diatoms were shown to be highly resistant to light-temperature stress and its associated photooxidative stress, thanks to their motility and to the physiological non-photochemical quenching (NPQ) of chlorophyll *a* fluorescence (Laviale et al., 2015).

The MPB biofilm is thus a unique transient biogeomorphological structure which constitutes a specific case study for *in situ* analyses of biological processes in surficial sediment. We investigated the dynamics and the activity of the viriobenthos associated with the MPB biofilm of the mudflat of Marennes-Oléron Bay (MOB; France) during the diurnal emersion period. The aims of our study were primarily, (1) to evaluate the temporal dynamics of viruses at the macro-(monthly) and at the micro-scale (hourly) and their horizontal distribution, and (2) to estimate the viral production and the virus-induced prokaryotic mortality. Secondly, we postulated that part of benthic viruses may also originate from microalgae and may interact with their dynamics. We confronted the *in situ* data with *ex situ* experimental values obtained from sediment surface layers containing motile MPB and inoculated with benthic and planktonic viruses in order to question the viral impact on both heterotrophic prokaryotes and microalgae with a focus on the photosynthetic productivity of MPB.

## Materials and methods

### Study site and sampling stations

Sampling was conducted at diurnal low tide, during the emersion period (4 h in length on average), on the mudflat located at the south end plume of the Charente estuary in Marennes-Oléron Bay (45°53′N 01°07′W, France). Intertidal mudflats represent 60% of the bay at low tide (Figure S1). MPB can migrate vertically through the fine muddy sediment particles (median grain size around 11 μm) and may rapidly cover between 80 and 90% of the top-surface of the sediment during the first half of the emersion. First, a 4 km cross-shore transect was surveyed at three stations (1, 2, and 4, Figure S1) on 5 March 2003, 18 June 2003, 30 September 2003, and 1 February 2004. Secondly, five hourly surveys were performed during the diurnal emersion period at station 2 in 2008. Three cores were taken from each 4-m^2^ quadra, randomly chosen in triplicate at each time-point within a 320 m^2^ study zone on the 19 and 20 February and 360 m^2^ on the 17, 18 and 19 July, few days before the spring tide on the 22 February and 21 July (for details, see Orvain et al., 2014a). Samples were also harvested for *ex situ* experiments (15 May 2009 and 3 May 2010). In May 2009, the correspondence Weight/Volume was estimated as 1.29 ± 0.02 g per ml of fresh sediment (*n* = 30), while the water content was 58.63% ± 1.55 (mean ± SD, *n* = 10; range 52–65). Given this water content, 1 ml of fresh sediment weighed 0.53 g after desiccation.

In each case, the 1 cm top surface sediment of three independent cores were sliced, pooled, and homogenized before sub-sampling in triplicate using 5 ml sterile syringe corers; they were then fixed with 4%-formaldehyde (V/V; 2% final concentration) and frozen (−20°C) 1 h later until analysis within a week. In parallel, subsamples may serve to acquire environmental data: salinity, Chl a concentration measured using a Fluorometer Turner TD-700, water-content estimated by drying (60°C for 12 h) and after a supplementary burning of 2 h at 490°C the concentration of organic matter (Table 1).

**Table 1:** **Environmental data and Virus to Prokaryotes ratio within the 1 cm-top surface sediment**.

	**Water-content (WC) (%)**	**Decrease in WC during emersion (%)**	**Change in pore-water Salinity during emersion (PSU)**	Organic matter Mass g g^−1^(%)^*^	Chl *a* μg g^−1^^*^	**Virus to Prokaryotes ratio (VPR)**
February 2008	61.7 ± 0.7	2.7	32–33	0.079 ± 0.001 (8.6%)	20.01 ± 0.45	0.85 ± 0.49
July 2008	51.3 ± 0.4	5.3–11.4	37–42	0.129 ± 0.007 (12.9%)	7.61 ± 0.23	9.61 ± 3.31
May 2009	52.6 ± 0.5	nd	nd	nd	nd	4.27 ± 0.08
May 2010	58.6 ± 1.5	nd	nd	nd	nd	1.89 ± 0.34

*Mean ± SD*.

* *per g of dry sediment*.

Water column samples were taken at the sub-surface at high tide on the same day, either on the vertical of station 2 (2003–2004 survey) or at station E (mouth of the Charente estuary; Auguet et al., 2005). Samples were fixed on board (36%-formaldehyde, 1% final concentration), stored at 4°C and analyzed within 6 h in the laboratory.

### Extraction of viruses

Benthic viruses were extracted, in triplicate. Briefly, 1.0 ml of tetrasodium pyrophosphate (10 mM final) and 3.6 ml of Milli-Q water were added to a slurry of 400 μl of fixed samples (i.e., 200 μl of fresh sediment) defrosted at 37°C, followed by 30 min of gentle shaking at 4°C on a rocking table and one centrifugation for 30 min at 1000 g. Use of ultrasounds (Danovaro et al., 2001) have been discarded after a first test, confirmed then by a comparative test (July 2011) by which the accuracy of our method was analyzed on three sediment samples, in triplicate, by comparison with the extraction method using probe sonication instead of shaking (Sonimasse S20, two periods of 30 s at 60W separated by 30 s of manual soaking).

This surfactant-procedure can occasionally be performed two or three times more with the pellet of the remaining settled sediment to test the efficiency of virus extraction, notably in February (*n* = 11) and July 2008 (*n* = 23), July 2011 (*n* = 9), and May 2013 (*n* = 12) and at each new sampling period in triplicate. The different supernatants, stemming from successive S-steps, were separately quantified immediately after recovery. Briefly, 2 ml of a final dilution of 20, 200, and 400 times in MilliQ-water (from the first to the third supernatant, respectively) were filtered through a 0.02 μm Anodisc 25 membrane (Whatman) and stained with SYBR-green I (Noble and Fuhrman, 1998). Slides were immediately enumerated for virus counts (15 fields) under a blue light (filter set 38, Zeiss) at x1000 magnification on a Zeiss Axioskop 2 Mot Plus epifluorescence microscope (Carl Zeiss, Inc.) with a 100x Plan APO oil objective lens. For the comparative test of method of extraction, the supernatants have been also quantified by flow cytometry according to the protocol of Brussaard et al. (2010); 10^−3^ dilutions were stained by Sybr-green I and 80°C heated for 10 min before the analysis using a FACSCanto II cytometer, calibrated with 0.47 μm beads and the FACSDiva software.

### Virus size and morphology

Viruses were first extracted in triplicate using pyrophosphate, a rocking shaker, and centrifugation as described above for the epifluorescence counts. Supernatants were pooled and ultracentrifuged for 3.5 h at 150,000 g (LE 70 Beckman ultracentrifuge, SW 28 rotor) and the pellet was resuspended in 100 μL of TN buffer (0.02 M Tris-HCl, 0.4 M NaCl, pH 7.4). The diversity in shape and size was analyzed by TEM. Two carbon-colodion coated grids (Cu/Pd grid, 300 mesh) were prepared per sample by negative staining using 2% phosphotungstate (Montanié et al., 2002). Observations were performed with a Jeol 2011 transmission electron microscope operating at 200 kV, calibrated with graphite grids, at a magnification of 50,000x to count at least 100 Virus Like Particles (VLP). Capsids were sized using Olympus analysis software.

### Prokaryotic enumeration

Triplicate samples (5 ml), defrosted at 37°C, were diluted 2000 times with 10 mM tetrasodium pyrophosphate at serial dilutions of 0.5, 10^−1^, and 10^−2^ [adapted from Pascal et al., 2009 and validated (Figure S2) by comparison with two other extraction methods using either cation-exchange resin (Lucas et al., 1996) or methanol Lunau et al., 2005]. A subsample was ultrasonicated for 30 s at 60 W (Sonimasse S20 sonicator), filtered onto a 0.2 μm black polycarbonate membrane, and the prokaryotic cells stained with DAPI (Porter and Feig, 1980) then enumerated under UV illumination (filter set 01, Zeiss) at 1000x magnification on a Zeiss Axioskop 2 Mot Plus epifluorescence microscope (100x Plan APO oil objective lens). In the text, bacterial and archaeal cells are indifferently grouped as prokaryotes.

### Microphytobenthos (MPB) counts

Homogenized mud (1 ml) was diluted 10-fold with <30-kDa ultrafiltered seawater (obtained by tangential flow through polysulfone cartridge (Montanié et al., 2014). Then 0.5 ml were diluted and fixed with 5 ml of alkaline lugol (1% final concentration). Algal cells were counted using a Nageotte chamber counter at x40 magnification on a Zeiss Axioskop microscope. MPB diatoms were divided into two groups: “small cells” which were mainly *Navicula* sp. and “large cells” which were mainly *Pleurosigma* and *Gyrosigma* spp., the so-called P-G taxon.

### *In situ* viral production and virus-mediated mortality of prokaryotes

*In situ* viral production was monitored during the emersion period as the net *in situ* viral abundance change by sampling, in triplicate (three independent cores), three randomly chosen quadras to evaluate the initial abundance and three other quadras to determine the change in viral abundance after 3 h of emersion. Samples were V/V fixed with 4% formaldehyde (2% final concentration) and frozen at −20°C. The lytic viral production (VP) was calculated as described by Luef et al. (2009) as the maximum minus the minimum viral abundance divided by the time elapsed. Virus-mediated mortality of prokaryotes (% of cell loss per time unit; Weinbauer et al., 2010) was deduced by dividing VP by the burst size (BS) as the number of lysed cells (VLC) and then by reporting it to prokaryotic standing stock (PSS), assuming a constant BS of 36 (mean value in Corinaldesi et al., 2010). Virus-induced mortality of prokaryotes (VIM, %) has also been evaluated as the ratio of lysed cells (VLC) to prokaryotic production (Danovaro et al., 2008a). The hourly carbon released by viral shunt from prokaryotes (VICR) was calculated assuming 79 fg C per cell (Saint-Béat et al., 2013) and weighted by the total Prokaryotic Carbon Demand (PCD) considering a bacterial growth efficiency of 31% in order to investigate the impact of viruses on C cycle (wVICR, Pinto et al., 2013).

### *Ex situ* experiment: viral lysis

To address and quantify the viral impact on benthic prokaryotes and microphytobenthos, only the free pore-water viruses were tested in order to avoid the time-consuming chemo-physical treatment during benthic extraction and to limit the input of mineral or organic matter detached from the biofilm. Pore-water viruses (“benthic viruses,” Vb) were harvested by centrifugation at 3500 g for 10 min (Jouan CR412) of fresh sediment (1 cm top-surface sediment), then filtered through a 0.2 μm membrane to eliminate all other microbes. Additionally, viruses in the water column (“planktonic viruses,” Vp) were isolated from other organisms by filtration of the overlying seawater through a 0.2 μm filter. In May 2010, heat-inactivated Vb (boiled and cooled 3 times) were tested.

Sub-samples of fresh sediment (6 × 2 ml) were incubated in 6-well microplates (Falcon), humidified top-down either with 250 μl of virus-free pore-water (“Control”) or 250 μl of benthic or planktonic viruses (“Vb or Vp treatment”). Virus-free pore-water was obtained by ultrafiltration of the virus-rich filtrate using a centrifugation filter device (Centricon Plus-70 Ultracel PL-30, Millipore). The 6-well-microplates were exposed to natural light at ambient temperature in order to maintain the *in situ* migratory behavior of MPB cells. Time-series sampling was performed daily in triplicate at the corresponding time of mid low tide in the field (using a 1 ml syringe corer after homogenization of the well, with a coefficient of variation of 13.41%). The impact of pore-water viruses on the prokaryotes was estimated over three consecutive days in May 2009 and 2010. Daily viral production and virus-mediated mortality of prokaryotes were calculated for the concomitant period of prokaryotic decrease and viral increase (Luef et al., 2009). Total prokaryotic loss was estimated as the net decrease in abundance (i.e., net prokaryotic production; Middelboe et al., 2006).

In May 2009, viral lysis activity of Vb was also evaluated on MPB over seven days and compared to Vp lysis activity. To counteract the possible evaporation of water from the sediment, 250 μl of virus-free pore water was added to each well at day 3.

### Photosynthetic activity of the MPB *ex situ*: Maxi-Imaging-PAM chlorophyll fluorescence measurements

Chlorophyll fluorescence measurements were performed with the Maxi-version of an Imaging-PAM chlorophyll fluorometer (I-PAM, Walz, Effeltrich, Germany) on a 6-well microplate, which occupies the total surface of the fluorescence image (10 × 13 cm; Figure S3). Three wells (one horizontal row) used for one kind of treatment only (control, Vb- or Vp-) enabled instantaneous triplicate measurements (Figure S3). The photosynthetic activity of the MPB was assessed by rapid light curve (RLC) measurements (Perkins et al., 2010). RLCs were obtained by the application of a series of 11 sequential short light exposures (20 s) with increasing irradiance from 0 to 1250 μmol photons. m^−2^ s^−1^. At each irradiance, $F_{m}^{\prime }$ and *F*_*t*_ were recorded. $F_{m}^{\prime }$, the maximum fluorescence yield, was measured by applying a saturation pulse (800 ms, 2800 μmol photons. m^−2^ s^−1^); *F*_*t*_, the steady-state fluorescence, was continuously monitored throughout each 20 s light step. $F_{0}^{\prime }$, the minimum fluorescence yield, was measured at irradiance 0 μmol photons. m^−2^ s^−1^ by measuring non-actinic light solely.

Two main parameters were computed from the RLCs: (i) ΦPSII, the effective quantum yield of photosystem II (PSII), was calculated for the 0 μmol photons m^−2^ s^−1^ irradiance as ΦPSII = ($F_{m}^{\prime }$–*F*_*t*_)/$F_{m}^{\prime }$. As no adaptation to the dark was performed before the measurement in order to avoid vertical migration of the motile microalgae, *F*_*t*_ (or $F_{0}^{\prime }$) and $F_{m}^{\prime }$ were close to their respective dark-adapted values *F*_0_ and *F*_*m*_, so that ΦPSII at this irradiance is close to the standard fluorescence index *F*_*v*_/*F*_*m*_, i.e., the maximum photosynthetic efficiency of PSII (Ralph et al., 2010), and (ii) NPQ, the non-photochemical quenching of chlorophyll fluorescence, was calculated as NPQ = $F_{m}-F_{m}^{\prime }$/$F_{m}^{\prime }$ (Ralph et al., 2010). The NPQ kinetics were further measured during a short (5 min) light exposure of 280 μmol photons m^−2^ s^−1^, which was close to the intensity necessary to saturate photosynthesis (249 ± 50 μmol photons m^−2^ s^−1^) for the control MPB biofilm, as measured using the RLCs.

### Statistical analysis

All statistics were performed with Excel and Prism 4 softwares or Minitab for nested ANOVA. Regression analysis was performed for prokaryote abundance and prokaryote loss against viral abundance and VPR, respectively, using log-transformed data.

## Results

### Efficient protocol for viral and bacterial extraction and counting from sediment

**Figure 1:**
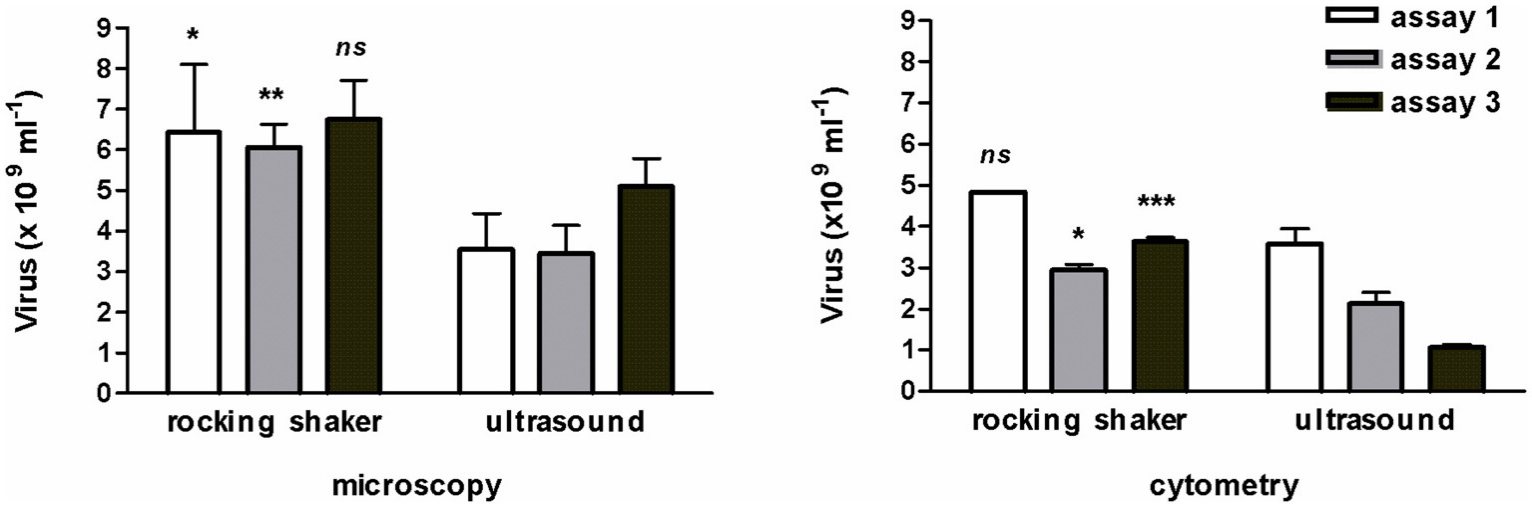
**Comparative test for viral extraction from muddy-sediment by shaking or sonication**. Reports of three assays performed in triplicate and analyzed by microscopy and cytometry (*n* = 3, mean ± SE). ns, non-significant, ^*^p < 0.05; ^**^p < 0.01; ^***^p < 0.001.

To extract viruses, sonication has been rejected because it emulsified the mud-samples and the sediment was disrupted into smaller particles that decreased the accuracy of the microscopic observation of viruses. Therefore, in the test of July 2011 (Figure 1), the microscopic abundance significantly lowered (ANOVA, *p* = 0.0002) as well as cytometric counts (ANOVA, *p* < 0.0001). The method of extraction (with or without sonication, nested ANOVA) accounted for 73.8% of total variance by microscopy and for 78.9% by cytometry. The accuracy of the protocol used for viruses (July 2011 test, *n* = 3) was illustrated by a coefficient of variation (CV) of 16.4 ± 8.4% instead of 19.3 ± 5.6% with the sonication step, and this difference in CV was confirmed by flow cytometry analysis (2.6 ± 1.9% vs. 13.3 ± 4.5%).

**Figure 2:**
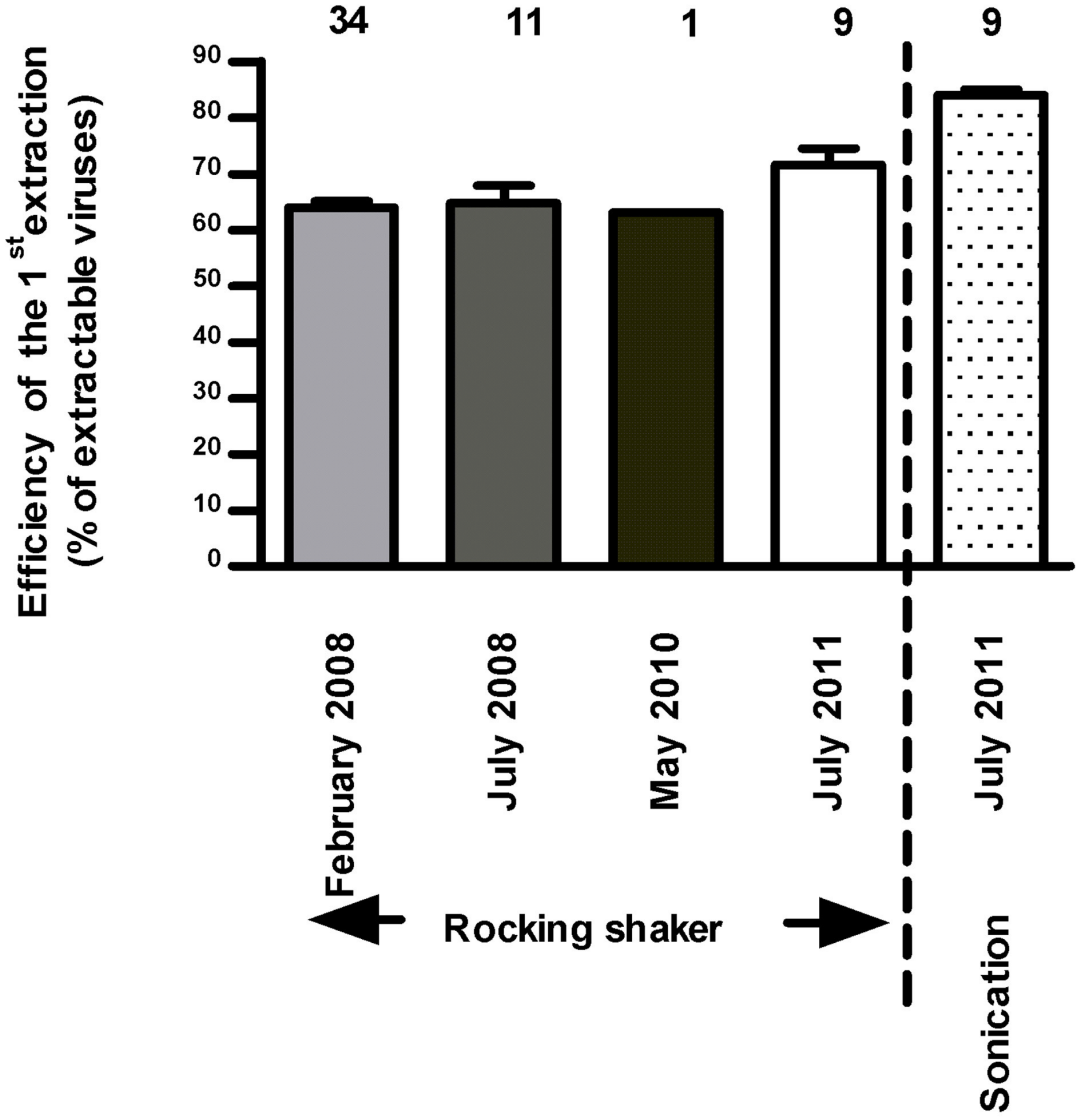
**Efficiency of the first extraction step of viruses (out of two) performed using rocking shaker and based on microscopic counts (*n* = 3, mean ± SE)**. Note that in July 2011, samples have been enumerated by microscopy and cytometry.

The addition of EDTA (Helton et al., 2006; Careira et al., 2015) was not conclusive for the same reason while vortexing has made the grids opaque for observation. After having tested our protocol on several extraction-steps, its efficiency seemed satisfactory. The first two steps (S1 and S2) involving pyrophosphate-shaking detected up to 99.6% of extractable viruses; a third step removed only 0.4% of the total cumulative number. The first and second steps extracted 63.9 ± 6.9 and 36 ± 1% (*n* = 34, *in situ* data of 2008) of the extractable viruses, respectively. Overall to date, all acquired data posteriorly validated that the first S1-extraction corresponded to 64.1 ± 8.1% of extractable viruses (*n* = 56, Figure 2). For samples of 2009–2010 after a preliminary confirmation of the percentage of extractability, only one step was performed for *in vitro* counts and the initial extractable virus numbers was then corrected for the miscounting based on the determined 64/36% ratio of S1/S2 extraction efficiency.

**Figure 3:**
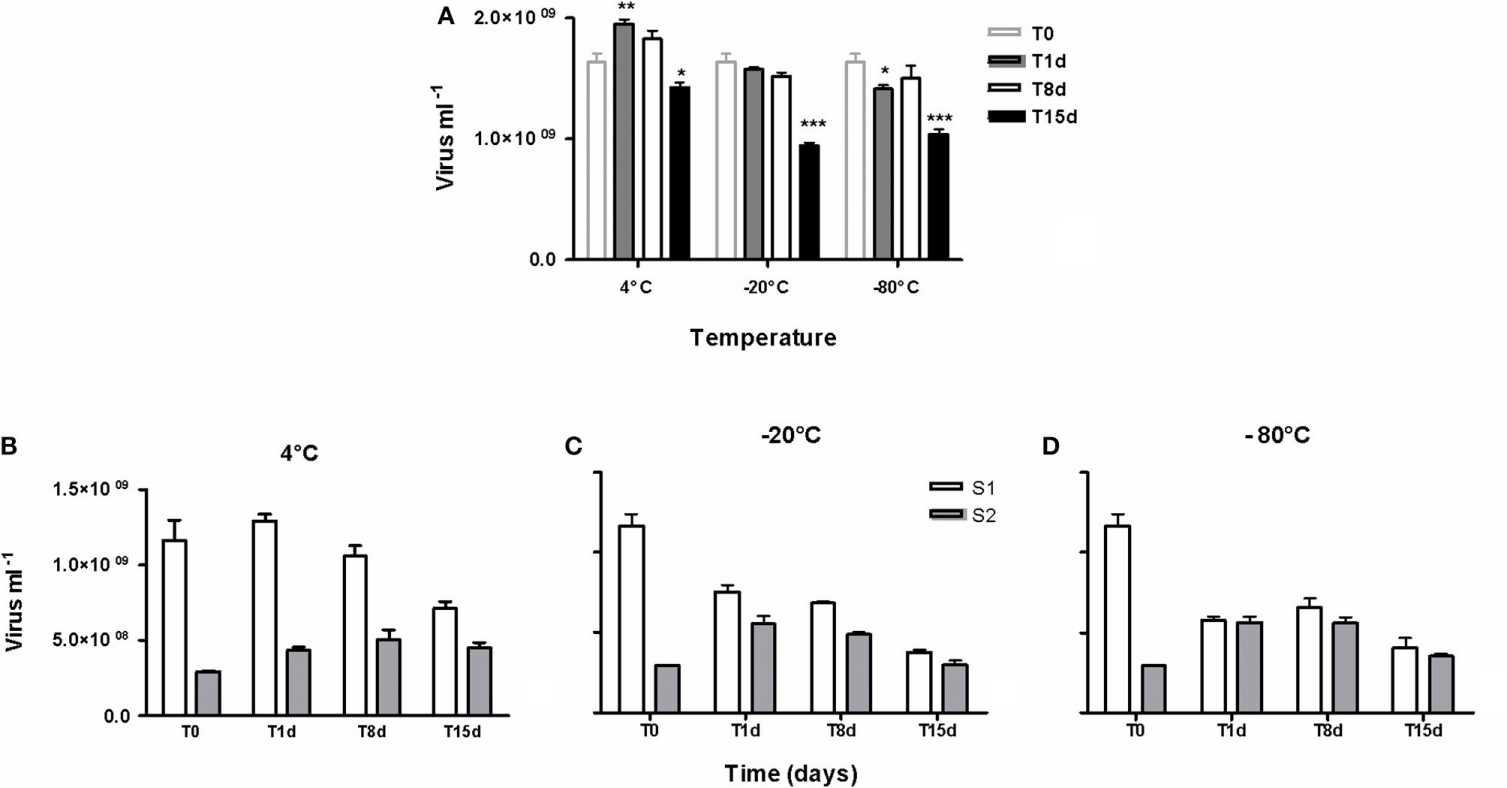
**Time-point persistence of viruses over 15 days during the preservation of sediment samples at 4°C, −20°C and −80°C. (A)** comparison of the persistent viruses at T1d, T8d, and T15d to the initial count (T0) based on counting by cytometry, ^*^p < 0.05, ^**^p < 0.01, ^***^p < 0.001. **(B–D)** Details of the efficiency of the two extraction-steps as numbers of viruses into the first and the second supernatants (S1, S2). (*n* = 3, mean ± SE).

All samples were stored at −20°C for a week and no factor was ever applied for correcting the viral loss due to fixation with formaldehyde and conservation. Indeed a time-point comparison of the decay of viruses included into muddy samples and stored for 15 days at 4°C, −20°C, and −80°C (Figure 3), revealed (i) no significant variation in virus counts after 8 days of storage at each or other temperature (*p* > 0.05), even if curiously the number of extracted viruses from samples stored at 4°C was higher after 1 day, (ii) irrespective of temperature, a significant loss of viruses between 8 d and 15 d of storage (*p* < 0.0001) and thus significant lowered *T*_15_ values compared to initial field value (*p* < 0.001) and (iii) at each time point, a higher preservation in refrigerated samples (4°C) than in frozen samples (−20°C and −80°C, *p* < 0.001) without any difference between the two freezing temperatures (*p* > 0.05). However, the percentage of S1 recovery declined over time in line with increased negative temperature of preservation (Figures 3B–D).

A significant fraction of the prokaryotes was not eluted with the extraction procedure used for viruses, despite three sequentially repeated extractions. Prokaryotes were thus counted independently from viruses with a protocol previously used in our laboratory for benthic prokaryotes (Pascal et al., 2009). Our protocol consists in ultrasound treatment of highly diluted sediments followed by a DAPI staining (Figure S2). The counting procedure was not disturbed by the accumulation of sediment particles inappropriately masking the microscopic slides.

### Monthly survey of viral and prokaryotic abundance

On a monthly average, the abundance of viriobenthos was 60-fold higher than that of virioplankton (60.3 ± 20.3, *n* = 7). The abundance of benthic prokaryotes varied between 244 ± 13 (May 2009–July 2008) and 1945 times higher (February 2008) than those in the water column.

**Figure 4:**
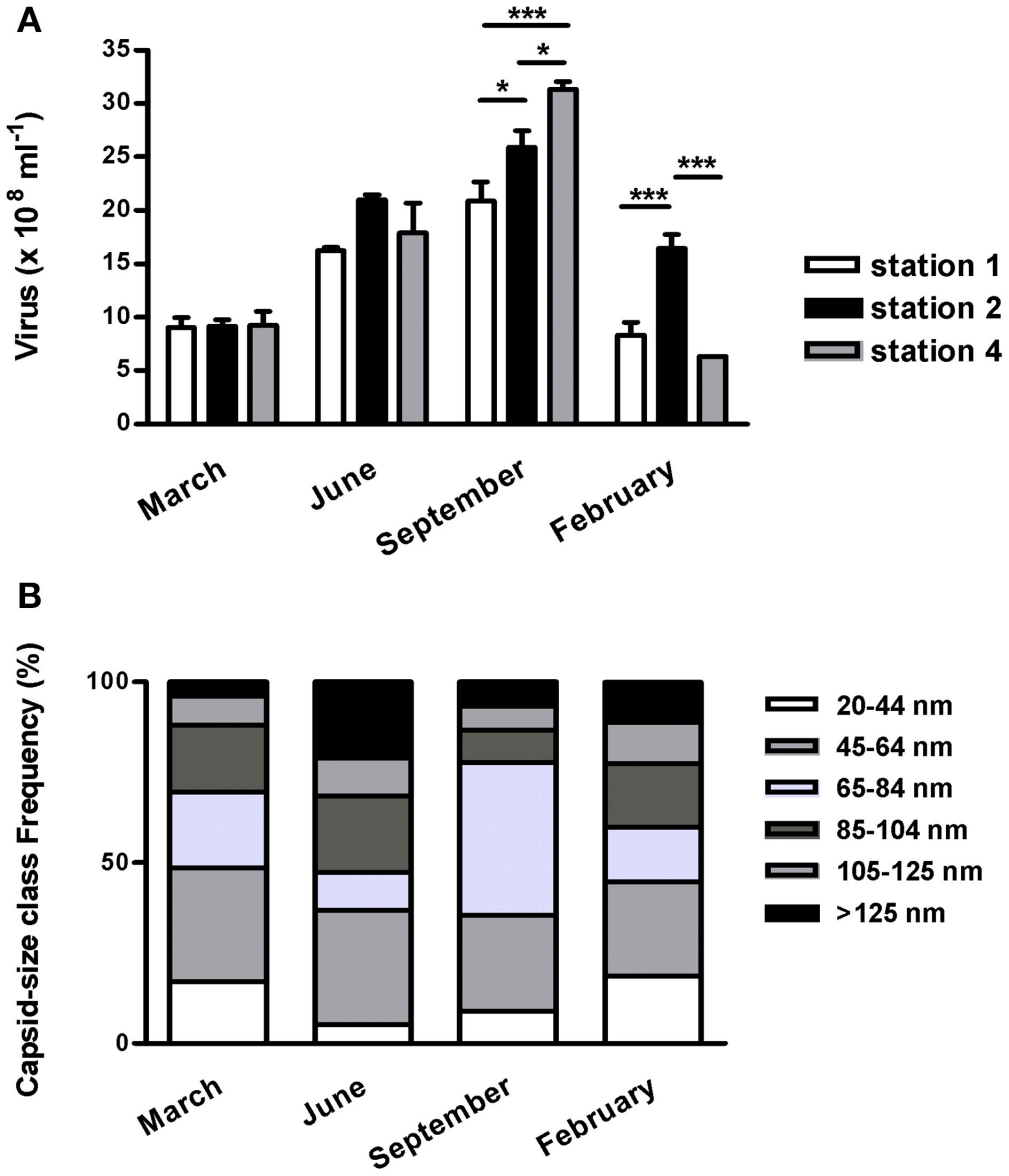
**Seasonal survey of viriobenthos at the diurnal emersion period of the Marennes-Oléron mudflat in 2003-2004. (A)** abundance along a 4 km cross-shore transect (stations 1–4; see Figure S1); mean ± SE. **(B)** Virus distribution by capsid class sizes; mean of the three stations surveyed. Only significant spatial heterogeneity between the three stations (Two ways ANOVA) were marked as ^*^p < 0.05, ^***^p < 0.001.

In 2003–2004, at all stations along the transect, viral abundance peaked to 2.43 ± 0.46 × 10^9^ viruses ml^−1^ in September then dropped to *c*. 0.9–1 × 10^9^ ml^−1^ in winter (Figure 4A). The sampling date accounted for 77.2% of the total variance in viral abundance while spatial location accounted only for 5.7%.

A large morphological diversity in virus-like particles (VLPs) was observed using transmission electron microscopy (Figure S4). The majority of VLP showed icosahedral shape, only few pleomorphic particles or elongated capsids were observed. Some filamentous forms of 0.6–1.2 μm were observed, notably in February and September (3–8%). Excluding filamentous VLPs, the capsid size was on average 77.4 ± 34.9 nm. Tailed viruses accounted for 4–21% of the particles and theirs capsids were on average 96.4 ± 25.7 nm in size. Because of the low frequency of tailed VLPs, their morphotypes were not discriminated between myovirus, siphovirus, or podovirus shape; however the largest particles could be address to Myoviridae. The distribution of capsid diameters provided accurate seasonal morphological comparisons (Figure 4B). Throughout the year, VLPs less than 65 nm in size constituted up to 36–49% of the total particles, depending on the frequency of the < 45 nm-sized particles (17–19% in winter vs. 5–9% in summer and autumn). The largest size-class (>125 nm) represented 4–21% of the total VLPs varying from 0.36 (March) to 3.85 × 10^8^ (June). In September, the 65–85 nm-sized particles was 5-fold higher than in the others months and predominated the viral community (42%). Tailed viruses accounted for 9–33% of their size-class. It was noticeable that, in February 2004, 22% of the VLPs were longer than 105 nm, and 38% of these were tailed viruses.

### *In situ* hourly survey of viral and prokaryotic abundance during an emersion period in winter and summer

**Figure 5:**
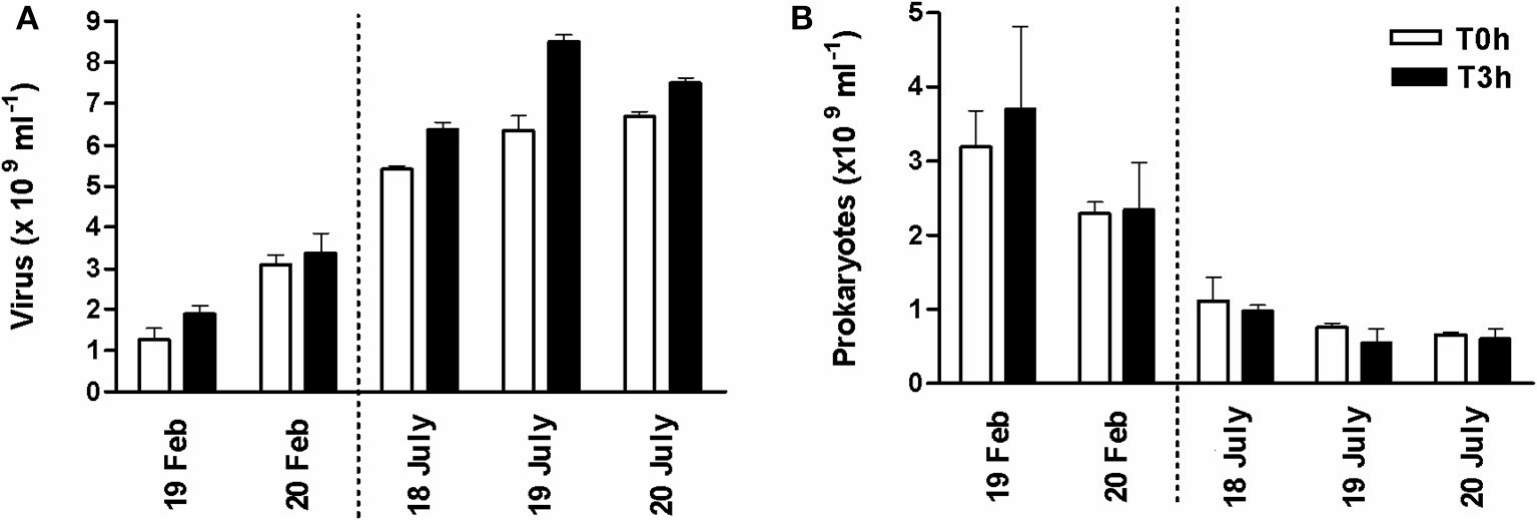
**Hourly survey of (A) viriobenthos and (B) prokaryotes during the diurnal emersion period in February and July 2008**. Abundance at the beginning of the emersion and 3 h later at station 2 of the transect (see Figure S1); mean of triplicate samples from three randomly chosen quadras ± SE.

In the top-surface sediment of the mudflat, viral abundance at the beginning of the emersion was *c*. 1.91 ± 0.22 × 10^9^ ml^−1^ in February 2008 and *c*. 6.30 ± 0.47 × 10^9^ ml^−1^ in July 2008 (Figure 5A). Prokaryotes numbers were 3.19 ± 0.45 × 10^9^ cells ml^−1^ and 8.48 ± 1.37 × 10^8^ cells ml^−1^, respectively (Figure 5B). Consequently, the Virus to Prokaryotes Ratio (VPR) was on average 0.85 ± 0.49 (*n* = 6) in February and 9.61 ± 3.31 (*n* = 8) in July (Table 1).

Viral abundance increased during the 3 h of diurnal emersion on the 19th and 20th February 2008 (+47% and 9%, respectively; Figure 5A) with no clear tendency for prokaryotes, resulting in an insignificant relationship between viruses and prokaryotes (*p* > 0.05). On average, the hourly viral production (VP) was 2.23 × 10^8^ viruses ml^−1^ h^−1^, and was responsible for 0.22 ± 0.04% of prokaryotic loss (in terms of prokaryotic standing stock, PSS). In summer, significant viral replication occurred on the 17, 18, and 19 July 2008 (*t*-test, *p* = 0.0037; +22.41 ± 4.74%) while prokaryotes concomitantly decreased (−5.44±1.97% h^−1^; Figure 5B). Viruses accounted for 84% of the variation in prokaryotic abundance (log Prokaryotes = −1.63 log Viruses + 24.90, *r*^2^ = 0.84, *n* = 6, *p* = 0.01). In July 2008, VP was 4.39 ± 1.42 10^8^ viruses ml^−1^ h^−1^, representing a loss of 1.52 ± 0.56% of PSS. Virus-mediated prokaryotic lysis accounted for 28.99 ± 5.61% of observed prokaryotic cell loss (i.e., the net prokaryotic growth). Moreover, considering that the gross prokaryotic production was 3.21 and 3.84% of the standing stock per hour (Production/Biomass; P/B), in winter and summer 2006 respectively (Pascal et al., 2009), virus-induced mortality (VIM) could account for 6.78 ± 1.44% of the prokaryotic production in February 2008 and for 39.7 ± 14.7% of prokaryotic production in July 2008. However, whatever the season, the viral turnover averaged 0.099 ± 0.082 h^−1^ (range 0.059–0.236), slightly higher in winter (0.14) than in summer (0.07). During the diurnal emersion, between 3.12 and 15.83 mg C m^2^ would be released by viral shunt per hour (i.e., VICR) and the released C may represent around 2.1% (winter) and 12.3% (summer) of the Prokaryotic Carbon Demand (i.e., weighted VICR).

**Figure 6:**
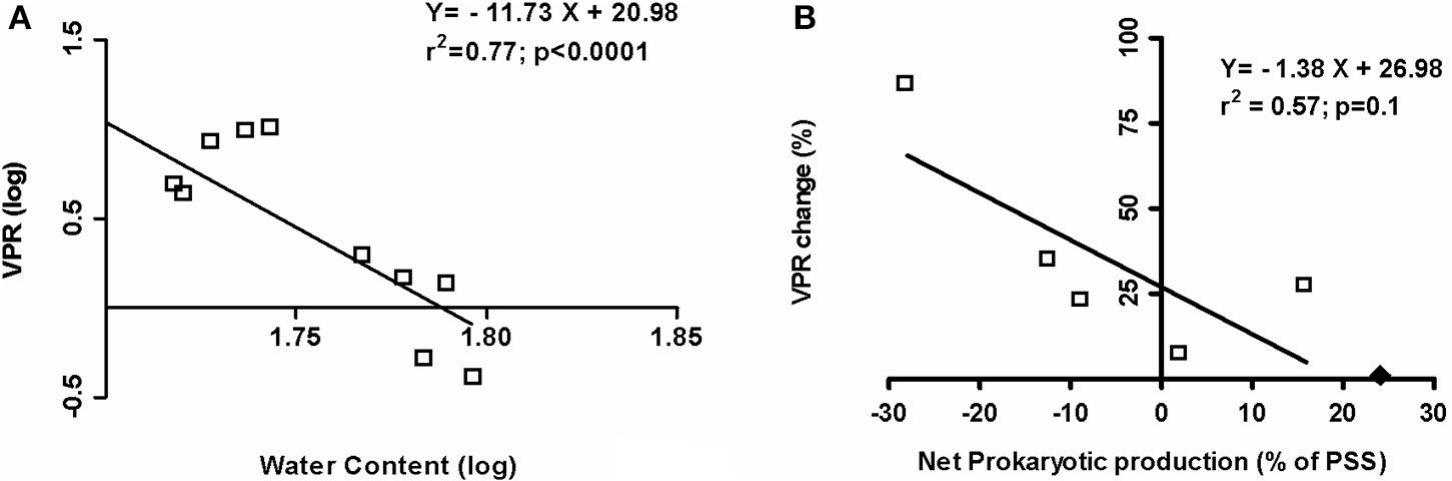
*****In situ*** Virus to Prokaryote ratio (VPR) in the top-surface sediment.(A)** Log/log relationship between VPR and the water content of the sediment, established with data averaged from triplicate samples taken in February and July 2008, May 2009 and May 2010 **(B)** Relationship between the variation of VPR during the 3 h of emersion and the net prokaryotic production (% of prokaryote stock standing) in February and July 2008. With ♦: outer data of 21 July 2008, the relationship would be: *Y* = −1.38*X* + 28.32 (*r*^2^ = 0.66; *p* = 0.05).

Interestingly, by using all the *in situ* data (February 2008, July 2008, May 2009, and May 2010), a significant negative relationship was observed between the virus to prokaryotic ratio (VPR) and the water content of the sediment (*p* < 0.0001; Figure 6A). A higher prokaryotic abundance was observed when the water content exceeded 58.6%, expressed *in fine* as a lower VPR (<2, February 2008 and May 2010 vs. 4.3 < in July 2008 and May 2009; Table 1). And during the emersion period and whatever the season, the viral production (VP) tended to be negatively linked to water content and the magnitude of change in VPR was positively and significantly related to viral production (*r* = +0.88; *p* = 0.01). When the net positive prokaryotic growth represented more than 19.5% of the PSS, VPR tended to decline during emersion (Figure 6B).

### Daily monitoring of top-surface spring sediments maintained *ex situ*: viral inoculation and virus-mediated prokaryotic lysis

In May 2009, in the top-surface mud layer, there were *c*. 2.81 ± 0.32 × 10^9^ ml^−1^ viruses and *c*. 6.58 ± 0.08 × 10^8^ ml^−1^ prokaryotes, corresponding to *c*. 5.30 ± 0.61 × 10^9^ and *c*. 1.29 ± 0.01 × 10^9^ g^−1^ dry sediment, respectively. The VPR was on average 4.27 ± 0.08. In May 2010, viral abundance was only 1.89 ± 0.34 times the prokaryotic abundance (*c*. 4.31 ± 0.19 × 10^9^ vs. *c*. 2.22 ± 0.07 × 10^9^ ml^−1^ or *c*. 8.13 ± 0.35 × 10^9^ vs. *c*. 4.18 ± 0.14 × 10^9^ g^−1^ dry sediment).

**Figure 7:**
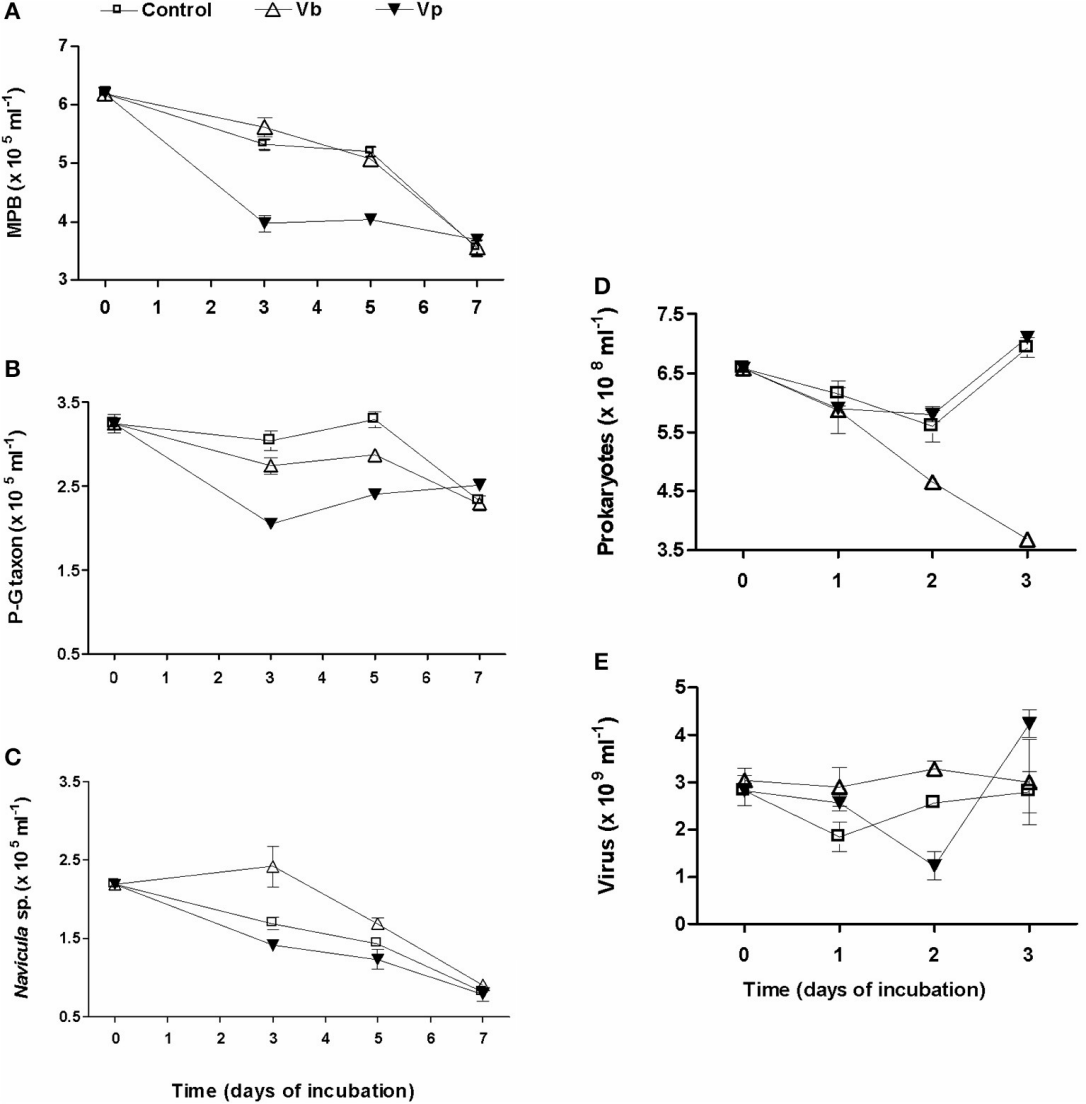
**Viral inoculation of top-surface sediment in microplates, in May 2009**. Virioplankton (Vp) and viriobenthos (Vb) were added and the two treatments were compared to the non-amended control. Time series over 3 days of the abundance of **(E)** viruses, **(D)** prokaryotes, and over 7 days of the abundance of **(A)** microphytobenthos with a focus on their dominant taxa, *Pleurosigma-Gyrosigma* taxon (P-G taxon; **B**), and *Navicula* sp. taxon **(C)**. Mean ± SE of three well-samplings.

In response to the addition of pore-water viruses (Vb) (+7.87% in 2009 and +3.84% in 2010), a daily increase in PSS loss was observed: +7.11% in 2009 and +11.69% in 2010. In 2009 (Figure 7E), viral abundance decreased during the first day, notably in control wells. A net viral production occurred during the second day while prokaryotic abundance was still decreasing. By day 3, viral and prokaryotic dynamics diverged between the control wells and virus-treated wells. In both cases, viruses rose back to their initial titers, while prokaryotes nearly doubled in controls compared to samples treated with viruses (Figures 7D,E). During the 3 days of incubation, the amplitude of variation in viral abundance was more pronounced in the controls than in virus-treated wells and thus was characterized by a higher VP: 4.73 × 10^8^ vs. 3.93 × 10^8^ viruses ml^−1^ d^−1^. However, during the first 2 days, viral abundance was significantly higher in virus-treated wells, leading to an increase of 1.07% in virus-mediated loss of PSS. Otherwise, no significant relationship was observed between viral and prokaryotic abundance (*r* = 0.32; *p* = 0.45).

**Figure 8:**
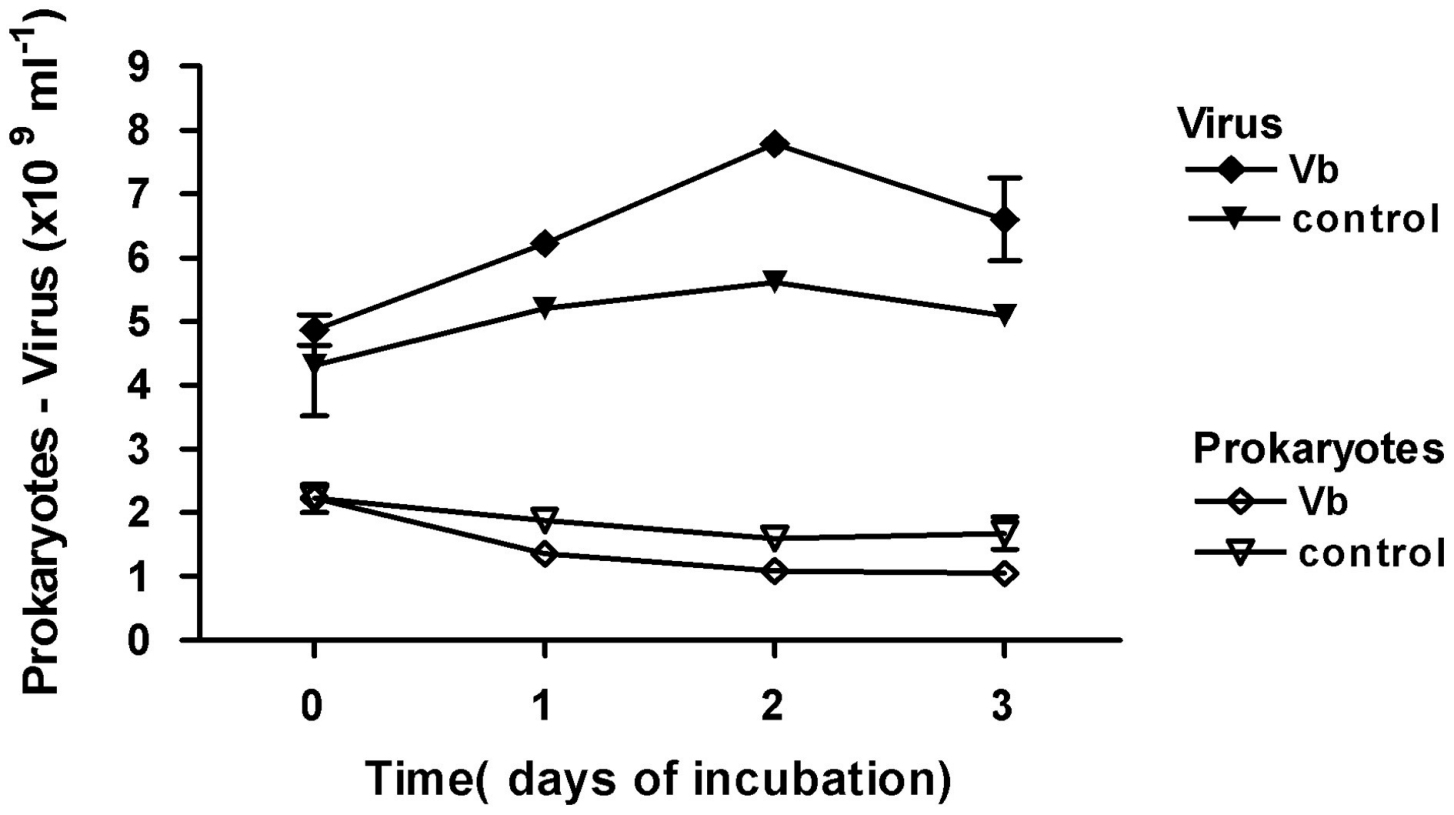
**Viriobenthos inoculation of top-surface sediment in microplates, in May 2010**. Viriobenthos (Vb) treatment was tested in comparison with the non-amended control. Time series over 3 days of the abundance of viruses and prokaryotes; mean ± SE of three well-samplings.

In contrast, in May 2010 (Figure 8), prokaryotic abundance decreased significantly with increasing viral abundance (log Prokaryotes = −1.46 log Virus + 23.43, *r*^2^ = 0.89, *n* = 8, *p* < 0.001) whatever the treatment. One day post inoculation, prokaryotic abundance was significantly reduced with the Vb treatment compared to controls (*t*-test, *p* < 0.05) while inactivated Vb had no effect (*t*-test, *p* = 0.5). The daily viral production, calculated over the first 2 days in control wells, was 6.48 × 10^8^ ml^−1^ d^−1^ of fresh sediment. In virus-treated wells, an additional viral production occurred, *c*. 8.09 × 10^8^ ml^−1^ d^−1^, which was responsible for the supplementary lysis of 1.01% of PSS. By combining the data from May 2009 and May 2010 with the *in situ* data from July 2008, a significant positive relationship between loss of prokaryotic abundance and VP was estimated as: prokaryotic loss = −0.759 × VP − 1.088 (*r*^2^ = 0.79, *n* = 5, *p* = 0.04).

In May 2009, the influence of Vp was also tested (Figures 7D,E). Only 6% of PSS was lost per day compared to 7.5% in the controls and 14.6% with Vb. However, daily VP reached 3 × 10^9^ particles ml^−1^.

### Daily monitoring of top-surface spring sediments maintained *in vitro*: MPB dynamics and photosynthesis as a function of viral inoculation

The analysis of the microphytobenthos (MPB) was only conducted in May 2009 (Figure 7). MPB biomass evolved similarly in the control and virus Vb-treated wells (Two way ANOVA, *p* = 0.363; Figure 7A). Of the 6.15 ± 0.11 × 10^5^ cells ml^−1^ in the top-surface sediment, pennate diatoms largely dominated over centric species (stable at around 12–13%, whatever the treatment), of which 59.6% belonged to the *Pleurosigma-Gyrosigma* taxon (P-G taxon) and 40.4 % to *Navicula* sp. In controls, a slight decline (3.1% per day) was noticeable until day 5, followed by a sharp decrease since only 57% of the original assemblage persisted at day 7 (Figure 7A). Within the first 3 days, the P-G taxon (Figure 7B) decreased slightly more (−9.8%) in the Vb-treated wells than in the control wells while the growth of *Navicula* sp. (Figure 7C) was significantly enhanced (+42.9 %, ANOVA, *p* = 0.045). From days 3 to 5, the P-G taxon biomass regained its level at *T* = 0 in control and, although a net increase was observed whatever the treatment, its biomass was significantly higher in the control wells than in the Vb-treated wells (+12.9%; ANOVA, *p* = 0.017). The abundance of *Navicula* sp. decreased by 30%, but the number of cells was still significantly higher in Vb-treated wells than in the controls wells (+18.7%, ANOVA, *p* = 0.04). At ay 7, no difference in abundance was observed whatever the treatment (ANOVA, *p* = 0.81 and 0.16 for the P-G taxon and *Navicula* sp., respectively). At that time, the loss in MPB biomass from T0 on was largely due to *Navicula* sp. (63%) while only 28% was due to the P-G taxon.

The addition of Vp significantly changed the MPB dynamics (Two way ANOVA, *p* < 0.001) by doubling the MPB decay rate to 6.9% d^−1^ vs. 3.1 and 3.6% for controls and Vb-treatment, respectively (Figure 7A). P-G taxon (Figure 7B) was significantly less represented than in the control wells over the 5 days (*p* < 0.008). In contrast, the number of *Navicula* sp. cells (Figure 7C) was only significantly lower at day 3 (ANOVA, *p* = 0.04).

After 5 days of viral infection, the photosynthetic activity of the MPB biofilm was measured with a Maxi-Imaging-PAM fluorometer. The basic chlorophyll fluorescence emission of MPB (*F*_*t*_), which illustrates the photosynthetic biomass, was similar in the control and virus-treated wells (Figure S3). This confirmed the results of MPB-cell counting. Nevertheless, the maximum photosynthetic efficiency (*F*_*v*_/*F*_*m*_) was clearly reduced in all virus treated-wells (Vb and Vp) compared to the control sediment (Table 2). It was noticeable that NPQ, the non-photochemical quenching of fluorescence, which is an index of light stress photoprotection, decreased in Vb-treated sediment but not in Vp-treated sediment, compared to the control (Table 2). Measurement of the NPQ development kinetics showed that not only the extent of NPQ was reduced but that the kinetic was slowed down by Vb infection (Figure S5). After 10 days of viral infection, the photosynthetic efficiency strongly decreased in all samples, illustrating the senescence of the MPB biofilm; there was no longer any difference between controls and virus-treated sediments (data not shown).

**Table 2:** **Photosynthetic parameters of controls (untreated) and virus-treated microphytobenthic biofilm after a five day infection**.

	**Control**	**Virus-treated Vb**	**Virus-treated Vp**
F_v_/F_m_	0.71 ± 0.01	0.65 ± 0.01	0.63 ± 0.01
NPQ	1.35 ± 0.01	1.15 ± 0.04	1.30 ± 0.03

*F_v_/F_m_ = the maximum photosynthetic efficiency of PSII. NPQ = the non-photochemical fluorescence quenching measured at a light intensity of 280 μmol·photons·m^−2^·s^−1^ which is known to saturate the photosynthetic electron transport rate (rETR) for the control biofilm (the light intensity required to attain rETR_max_, E_m_ = 249 ± 50 μmol·photons·m^−2^·s^−1^). Values are means ± SD of three measurements*.

## Discussion

### Methodological considerations

Comparative storage of sediment samples allows to conclude to a relatively good persistence of viruses at short-term (a week) at 4°C as well as freezing temperatures. While the loss of viruses may be up to 35% in 24 h (according to the exponential model of decay, Wen et al., 2004), a gain of viruses (+19%) was detected in 24 h at 4°C like in the case of lacustrine sediment (up to 31% in 5 days, Duhamel and Jacquet, 2006). Changes in the structure of the sediment matrix (organic matter and silt-clayed substratum) may modify the extractability of the viruses and their sorption capacity to the plastic tube-wall according to the extended-DLVO theory (Helton et al., 2006; Gutierrez and Nguyen, 2012; Wong et al., 2013). The apparent higher viral counts at 24 h may be consecutive to a stark release in pore water of colloidal EPS from microalgae and bacterial cells. EPS may help to desorb viruses by reducing the minimum energy depth and increasing steric hindrance (Walshe et al., 2010; Zhao et al., 2014) and then favor their persistence by inhibiting the extracellular nuclease or protease (Hewson et al., 2012). They may finally limit the bias of sorption on the plastic tube-wall of tube by complexing viruses in solution (Wong et al., 2013). Forehand, the recurrent loss of viruses into frozen samples compared to refrigerated samples may result from the disruption of cells due to ice crystal disruption (Helton et al., 2006) and the subsequent release of virucidal substances. Inversely no differences were reported for sediments of the Chesapeake Bay that contains greater proportion of sand and smaller percentage of organic matter (Helton et al., 2006).

Roughly, 64% of the extractable viruses were dislodged during the initial extraction step. This is similar to values reported for estuarine sediments from other sites (Helton et al., 2006; Danovaro and Middelboe, 2010). Given the physical properties of the sediment, an ultrasound treatment was recommended by Danovaro and Middelboe (2010) and Careira et al. (2015). Nevertheless, in our case, the presence of very small silt-clayed particles (up to 98%; Blanchard et al., 1997) precluded the use of a physical treatment (ultrasound and vortex). Indeed, compared to sandy sediment, the higher total porosity and lower permeability of muddy sediment favors the enhancement of the electrostatic forces between clayed particles and virions (Gerba, 1984; Helton et al., 2006) and a slow desorption of viruses (Pinto et al., 2013). Although, the adaptation of the extraction protocol we propose here for highly clayed sediments can be considered as conservative for viral extraction, it could not be validated for the extraction of prokaryotes.

Given the intertidal nature of the Marennes-Oléron bay (MOB), we made improvements to the methodology for the determination of viral production (VP). Estimates of viral increase over time is standard for marine sediments either by incubating (1) undiluted and homogenized deep-sea sediments in a Würgler-bag in anoxic conditions, (2) diluted slurry similarly to a pelagic analysis (Glud and Middelboe, 2004; Danovaro et al., 2008a; Corinaldesi et al., 2010) or by maintaining (3) intact Haps-cores of coastal sediments in water (Siem-Jørgensen et al., 2008). However, both the dilution-based and Würgler-bag approaches (1 and 2) suffer from methodological biases altering either the heterotrophy activity and the mineralization rate, the host-virus contact and progeny of infections or the loss of viruses by exoprotease (Hansen et al., 2000; Danovaro et al., 2008b; Dell'Anno et al., 2009). Although, the dilution-based technique is recommended by (Dell'Anno et al., 2009) as the most suitable methodology to estimate VP in marine systems, we chose to deduce VP directly in the field, during the emersion period, from net temporal variations in viral abundance as reflecting the true *in situ* production of surface sediment. By mimicking the mudflat in a low-tide situation, the microplate approach, is quite similar to the Würgler-bag method because it includes a homogenization step to uniformly distribute the undiluted mud into the wells. It reposes upon the same assumptions in terms of heterotrophic prokaryotes activity, biocide activity and competition with predators. Additionally, “Microplate incubation” is compatible with the use of Imaging-fluorometers to simultaneously study photosynthesis. To our knowledge, this is the first time the Imaging-PAM (I-PAM) has been applied for the assessment of the effect of viral infection on the photosynthetic activity of mudflat MPB natural assemblages. As a non-destructive technique and a rapid assay, I-PAM greatly facilitates measurements on complex samples collected *in situ* and maintained *ex situ* and allows the accurate implementation of photosynthesis regulation kinetics.

### Are benthic viruses mainly prokaryotic phages or eukaryotic viruses?

Viral abundance in the mudflat of the MOB is within the range reported for marine sediments (from 10^7^ to 10^11^ ml^−1^; Helton et al., 2006) and is even closer to the results reported for freshwater and shallow marine ecosystems (9 × 10^9^ viruses g^−1^; Danovaro et al., 2008b). The viral abundance of mudflat sediments was 60-fold higher than in the overlying water column. Such small ratios have been reported for other eutrophic bays: x22 for Moreton Bay (Hewson et al., 2001a), x14 for Niva Bay (Middelboe et al., 2003), and x10 for Chesapeake Bay (Helton et al., 2006) while higher values (from 100 to 1000) were observed in oligotrophic sites (Hewson et al., 2001a; Danovaro et al., 2008b). Because of a much higher abundance of prokaryotes in mudflat sediment, the virus to prokaryote ratio ranged 0.8–9.6 like for Dutch intertidal sediment (0.6–1.4; Careira et al., 2015); benthic VPR was lower than previous observations in the MOB water column (11.6 ± 3.7 in 2002–2003; Auguet et al., 2005). This general trend (except across the mouth of Chesapeake Bay; Drake et al., 1998) suggests a low viral production from prokaryotes in the sediments, even though the high density of prokaryotes and viruses probably fosters host-virus encounters (Filippini et al., 2006) which in turn may enhance prokaryotic resistance (Weinbauer et al., 2009). This situation is even more striking in that the higher availability of nutrients and organic matter in the sediment favors a higher activity of benthic heterotrophic prokaryotes (Danovaro and Serresi, 2000). This discrepancy could be explained by several factors that may be inferred from the virus-prokaryotes interaction: (i) different viral decay due to nuclease and/or protease concentrations (Middelboe et al., 2003; Filippini et al., 2006; Dell'Anno et al., 2015); (ii) a possible sorption on mineral matter or embedding in the EPS matrix, limiting the movement of bacteria and viruses and/or masking the viral receptors of bacterial cells (Danovaro and Serresi, 2000; Filippini et al., 2006), although polysaccharide depolymerases on viral capsids are known to degrade the EPS matrix (Sutherland et al., 2004); (iii) a hypothetical prevalence of lysogeny or chronic multiplication (Middelboe et al., 2003; Danovaro et al., 2008b); (iv) a reduction in the probability of virus-sensitive hosts encountering due to both high viral (Hewson and Fuhrman, 2007; Helton and Wommack, 2009) and bacterial diversities (Torsvik et al., 2002); and (v) a direct influx of viruses from the water column which settle, or indirectly as a result of the settlement of lysogenic prokaryotes and/or cells visibly infected by lytic viruses (Hewson and Fuhrman, 2003; Taylor et al., 2003; Danovaro et al., 2008b; Pradeep Ram et al., 2009).

The autochthonous or allochthonous origin of benthic viruses is still a matter of debate. Some evidence supports an endogenous origin without excluding an input of pelagic viruses (Siem-Jørgensen et al., 2008). However, in the study case of the microphytobenthic (MPB) biofilm, the proportion of phytoviruses may be significant or even it may oversize the proportion of prokaryotic phages among the viriobenthos. The high abundance of viruses in the surface sediment without any sign of intensive viral infection of prokaryotes (low VPR) may thus originate (1) in the sorption of large particles, algal viruses, from the water column and/or (2) in the replication through benthic microalgae, all the more so since the burst-size of algal viruses (range 10^2^–10^4^; Short, 2012) exceeds those of prokaryotes (range 3–69 in marine sediments; Danovaro et al., 2008b). Our results are congruent with both hypotheses. Indeed in the MOB intertidal mudflat, only 50% of viruses had a capsid size of less than 65 nm compared to 71% in the overlying water (Auguet et al., 2006). The sorption of viruses and the binding links on clay- and silt-particles enhances proportionally to the capsid-size (Dowd et al., 1998; Chattopadhyay and Puls, 1999). Moreover, large-sized virus particles may strongly counteract against the forces of desorption when the organic matter increases during emersion and the ionic strength decreases at rising tide (Gerba and Schaiberger, 1975). Overall, our results support the scenario of the replication of planktonic viruses through MPB diatoms since the addition of planktonic viruses (Vp) only slightly changed the daily loss of benthic prokaryotes but significantly declined diatom microalgae biomass. This enhanced viral production at the expense of MPB diatoms, as a result of input of pelagic viruses, may suggest fluxes of viruses at the water-sediment interface via the MPB biofilm. Nevertheless, we clearly demonstrated the negative impact of benthic viruses on prokaryotes since changes in VP explained 79% of the changes in net prokaryotic growth, even though this was delayed compared to VP (power slope = 0.75). This delay sustains the idea of a related viral replication through MPB hosts, in line with the relative high frequency of large capsid-sized virions while among the algal viruses isolated to date, virions size ranged from 22 to >200 nm (Short, 2012).

### Viral production and prokaryotic mortality

The value of 10^7^–10^8^ viruses produced ml^−1^ h^−1^ is in the range of 10^6^–10^9^ viruses g^−1^ h^−1^ reported for marine sediments (Danovaro et al., 2008a; Corinaldesi et al., 2010). Like in the deep sea sediments of Sagami Bay (Middelboe et al., 2006), VP may be responsible in mudflat for an average of 29% of the net bacterial losses. Cell loss of 0.2–1.5% of the PSS and virus-induced mortality of 7–40% of prokaryotic production per hour, confirm the ascending gradient in viral-induced prokaryotic mortality in terms of production from coastal sediments (around 16%, e.g., 12–57% in Adriatic Sea, Mei and Danovaro, 2004 and 4–41% in Central Øresund, Denmark, Siem-Jørgensen et al., 2008) to deep-sea sediments (89%, Danovaro et al., 2008a) and positioned MOB mudflat in terms of viral impact on prokaryotic standing stock together with the sites with the lowest cell losses (0.3%: Adriatic sea, Mei and Danovaro, 2004); 0.08–6.7%: Central Øresund, Denmark (Glud and Middelboe, 2004; Siem-Jørgensen et al., 2008); < 1%: deep-sea sediments (Middelboe et al., 2006). The highest cell losses were reported in Southern California (4–14%; Hewson and Fuhrman, 2003). Although, there was little apparent involvement of viruses in prokaryotic mortality, the net prokaryotic loss (the so-called prokaryotic net production) was positively related to the viral production rate in MOB during the emersion period. Differences in virus origin have been related to amplitude in prokaryotic responses; prokaryotes appeared more permissive to interstitial viruses (extracted from fresh sediment) than to surficial pelagic viruses. Benthic prokaryotes would mainly be of benthic origin and their viruses too. Our bioassays confirmed that after erosion into the overlying water, benthic bacteria and their viruses would be mainly located at the water interface and not dispersed efficiently upwards in the entire water column (Guizien et al., 2014).

### Impact of viruses on the MPB diatoms

In MOB, MPB diatoms may be easily eroded from mudflat and 30–80% of diatoms of the water column can be from benthic origin depending of hydrodynamics (Guizien et al., 2014). Also there is a strong relationship between planktonic viruses and phytoplankton (Ory et al., 2010). Microalgae and especially diatoms have already been described as potential hosts for viral multiplication in the estuarine benthos and phytoplankton (Hewson et al., 2001b), and in monospecific cultures (planktonic diatoms, Nagasaki, 2008; Tomaru et al., 2012); benthic diatom *Navicula* sp. (Suttle et al., 1990). Thus, in MPB biofilm associated to surficial sediment, both prokaryotic, and algal viruses were logically present. Indeed for Chesapeake Bay benthic virome, 11% of the viral hits to dsDNA belonged to the *Phycodnaviridae* family (Helton and Wommack, 2009).

As a consequence, the impact of viruses on MPB is more complex than on prokaryotes, due to higher dispersion of diatoms in the entire water column. Planktonic viruses like benthic viruses may regulate diversity and photosynthetic activity of the MPB. In our experiments, during the first days of infection, Vb modified the abundance of MPB diatoms by promoting the growth of *Navicula* sp. while inhibiting the growth of the P-G taxon, the two dominant taxa. In contrast, Vp decreased the abundance of both taxa, with a greater impact on the P-G taxon, which thus appeared to be more sensitive to viral infection (by both Vb and Vp) than *Navicula* sp. This observation is congruent with the diatom species specificity of viruses (Nagasaki et al., 2005) and may infer a selective growth inhibition of a subset of the microalgal community (Castberg et al., 2001; Hewson et al., 2001b; Larsen et al., 2004). Some microbial/diatom species may then benefit from the subsequent lower competition for light and nutrients.

Before the lysis of cells, the selective decline of a community or a population is probably linked to the negative effect of viral infection on photosynthetic activity, as reported before for phytoplankton (Suttle, 1992; Hewson et al., 2001b; Juneau et al., 2003). Indeed, both Vb and Vp infection of MPB decreased its photosynthetic efficiency (*F*_*v*_/*F*_*m*_). Additionally, Vb infection specifically decreased and slowed down the development of the photoprotective NPQ; this is in contrast to the effect of Vp infection in the bloom-forming raphidophyte *Heterosigma akashiwo* (Juneau et al., 2003). Interestingly, light intensity and UVB radiation are important factors controlling algal host-virus interactions (Jacquet and Bratbak, 2003; Baudoux and Brussaard, 2008). This is even more significant since it is well documented that the diatom and MPB communities have a powerful NPQ and that in reaction to a decrease in NPQ, MPB photosynthesis and behavior are impaired (Laviale et al., 2015). The decrease in NPQ could render the cells more sensitive to environmental stresses, i.e., high light, temperature and salinity stresses (Juneau et al., 2015; Laviale et al., 2015). Forehand for virus-infected plant models, NPQ may be a relevant “disease signature” to diagnose the different stages of infection, increasing locally at the early stage of viral infection and decreasing at the final stage in senescent tissue (Pérez-Bueno et al., 2006; Pérez-Clemente et al., 2015). Further, studies will be useful to extrapolate the virus biotic effect on NPQ to photosynthetic protists.

In this context, Vp generated the highest Viral Production (VP) together with the highest impact on MPB biomass and photosynthesis but had no effect on NPQ. Despite the reduction in photosynthetic potential, the maintenance of photoprotection may support the permissiveness of cells (or sub-sets of cells) by offering a sufficient energy level for viral replication (Juneau et al., 2003; Baudoux and Brussaard, 2008). In contrast, the viral yield during Vb infection may be limited by the decrease in the photoprotection capacity of MPB. These observations support the hypothesis of Baudoux and Brussaard (2008) that diatom species-specific photo-acclimation/-protection capacity (defined according to their habitat of origin, Barnett et al., 2015) may determine the differential effect of irradiance on viral propagation by influencing the burst size and/or the latent period. Overall, efficient Vp infection of MPB questions the real susceptibility of MPB diatoms to viruses in the sediment and in the water column due to their upward sediment-water transport at high-tide (i.e., resuspension in the water column) and their downward water-sediment transports when settling.

### Ecological implications

The MPB biofilm of intertidal mudflats is a product of complex interactions between microalgal primary producers, bacteria and viruses. The specific algae-prokaryotes coupling, as well as the structure of the prokaryotic community and its remineralisation activity (Glud and Middelboe, 2004; Haynes et al., 2007; Danovaro et al., 2008b) have been related to (i) the availability of labile organic matter derived from detritus (Galois et al., 2000), (ii) the cell-derived EPS production (Haynes et al., 2007; Bruckner et al., 2011), and (iii) the virus-mediated production of DOM as cellular debris and decomposed virions (Wilhelm and Suttle, 1999; Sutherland et al., 2004; Dell'Anno et al., 2015).

Pore-water content would be one of the main factors determining the encounter rate between viruses and hosts (Weinbauer et al., 2009). For the MOB mudflat, as in soil (Srinivasiah et al., 2008), water content (WC) was inversely correlated with the Virus to Prokaryote Ratio while Pinto et al. (2013) reported a positive relationship from global analysis of worldwide *in situ* data and WC was positively related to prokaryotes abundance. Nonetheless at the emersion-scale, the VPR always varied inversely to net bacterial growth, from negative to positive values, since a net viral production occurred during emersion concomitantly to the decrease in water content and in line with the negative links between porosity and VP (Pinto et al., 2013) and viral abundance (Helton et al., 2006). No change of the VPR over emersion occurred when the net prokaryotic increase was around 20% of PSS. Interestingly, we observed such similar features in the dynamics of the viruses and prokaryotes, at spring tide on July 21 of 2008 (outer data on Figure 6). This was a singular day characterized by a minimum value of Chl *a* biomass and a high erodibility, which may be partially explained by the destabilizing effect of a more pronounced hygroscopic feature of EPS (see for details, Orvain et al., 2014b). Therefore, the occurrence of area of water retention and the breaks of cell-matrix bonds may corollary favor *in fine* the bacterial growth. However taking into account the viral dynamics and the VPR allows us to also postulate that phytophages may be responsible of the observed decline of MPB on July 21 supporting indirectly the bacterial growth. To round off this item, VPR may be a good integrative proxy for the description of the functioning of the microbial food-web within a complex biofilm. It reflects both the interactions between the different microbial components (virus, prokaryotes, MPB), and their respective and interlinked relationships with water content and the bioavailability of organic matter but also its hydrophobicity (notably the protein/polysaccharide ratio of EPS).

Like in water column where viral abundance is influenced by the quality, size and age of the aggregates (Weinbauer et al., 2009), it may be related, in the case of intertidal mudflat, to the maturation and the structure of MPB biofilm, which is seasonally distinguishable by differences in the bioavailability of the organic matter (as detailed in Orvain et al., 2014a). Briefly, DOM was higher in the developing biofilm of July 2008 and its composition may traduce a synergetic collaboration between highly active diatoms and prokaryotic cells in the resistance to strong irradiance and salinities whereas the algal biomass and prokaryotic abundance standing stock were less abundant than in the more stabilized biofilm. In winter 2008, diatoms excreted bound EPS carbohydrate enriched in rhamnose that can promote the biostabilization of the sediment and act as bacterial development sensor (Pierre et al., 2012; Orvain et al., 2014a). Moreover, the dense population of the snail *Peringia ulvae* in summer may also infer seasonal differences in microbes abundance due to grazing activity (Orvain et al., 2014b) and/or vertical bioturbation of sediment (as proposed for subpolar ecosystem, Wróbel et al., 2013). Nevertheless, we can postulate that, during the ingrowing of biofilm (July), the viral production was enhanced since both microalgae and prokaryotes were metabolically active (prokaryotic P/B = 3.84) without allowing, nevertheless, an efficient viral turn over. On contrary, when the MPB biofilm was better structured (February) but less active (prokaryotic P/B = 3.21), the prokaryotes and the microalgae grew under steady-state conditions in phase with a lower but more efficient viral production to maintain the viral stock.

Overall, this study credited the previously report of seasonal variation of the benthic viral shunt and the estimated supplies for Prokaryotic Carbon Demand (PCD), i.e., 2 and 12% of PCD, in winter and summer respectively, considering all viruses as prokaryotic phages (to be compared to 0.1–10% of PCD; Pinto et al., 2013). Therefore, the impact of viruses may appear negligible for nutrition of heterotrophic prokaryotes in surface sediment worldwide compared to deeper anoxic sediment (30%, Danovaro et al., 2008a).

## Concluding remarks

Mudflat viriobenthos is a highly active component of the MPB biofilm during emersion. Viral infections play an important role in the functioning of the surficial sediment of intertidal mudflat with a seasonal variability in the viral mediated mortality of prokaryotes. However, a sizeable part of benthic viruses (and probably of pelagic viruses) originates from MPB and may regulate biomass and diversity of the benthic diatoms/microalgae. Therefore, viruses must be included to current models of the functioning of the benthos-pelagos coupled food-web of intertidal mudflats not only as bacteriophages (Saint-Béat et al., 2014) but also as phytophages albeit the partitioning between the phages of MPB and prokaryotes remains to circumscribe, as well as the exact impact of benthic and planktonic viruses on MPB and phytoplankton biomasses.

### Conflict of interest statement

The authors declare that the research was conducted in the absence of any commercial or financial relationships that could be construed as a potential conflict of interest.
