# Supplementary material for: Viral Impact on Prokaryotic and Microalgal Activities in the Microphytobenthic Biofilm of an Intertidal Mudflat (French Atlantic Coast)
Source: Front Microbiol. 2015 Nov 10;6:1214. doi: 10.3389/fmicb.2015.01214 (PMC4639598; doi:10.3389/fmicb.2015.01214)
Supplement: Supplementary file 1 [file Image1.PDF]

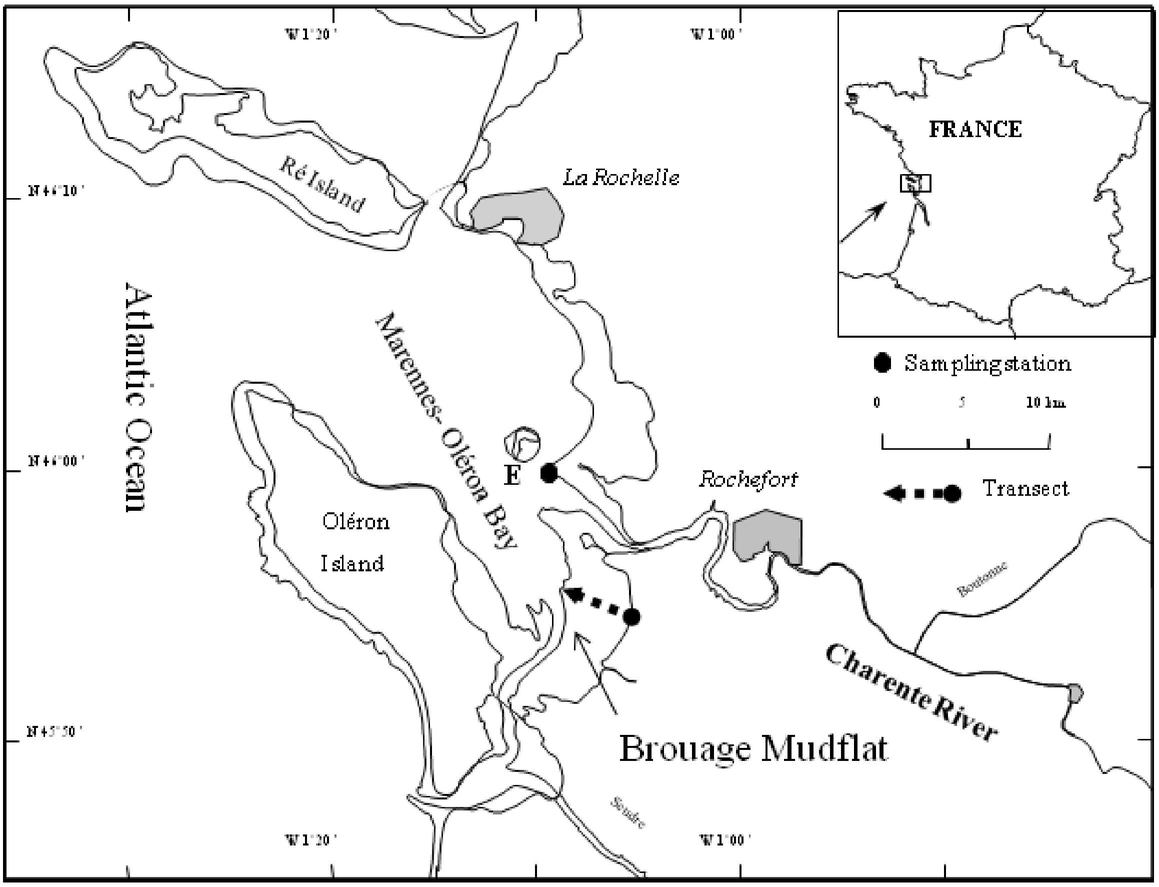

Fig. S1. Map of the cross-shore transect on the Brouage mudflat in Marennes-Oléron Bay. Note the point E (for water sampling) located at the outer estuary of Charente River and the transect on the mudflat.
