# Supplementary material for: Viral Impact on Prokaryotic and Microalgal Activities in the Microphytobenthic Biofilm of an Intertidal Mudflat (French Atlantic Coast)
Source: Front Microbiol. 2015 Nov 10;6:1214. doi: 10.3389/fmicb.2015.01214 (PMC4639598; doi:10.3389/fmicb.2015.01214)
Supplement: Supplementary file 2 [file Image2.PDF]

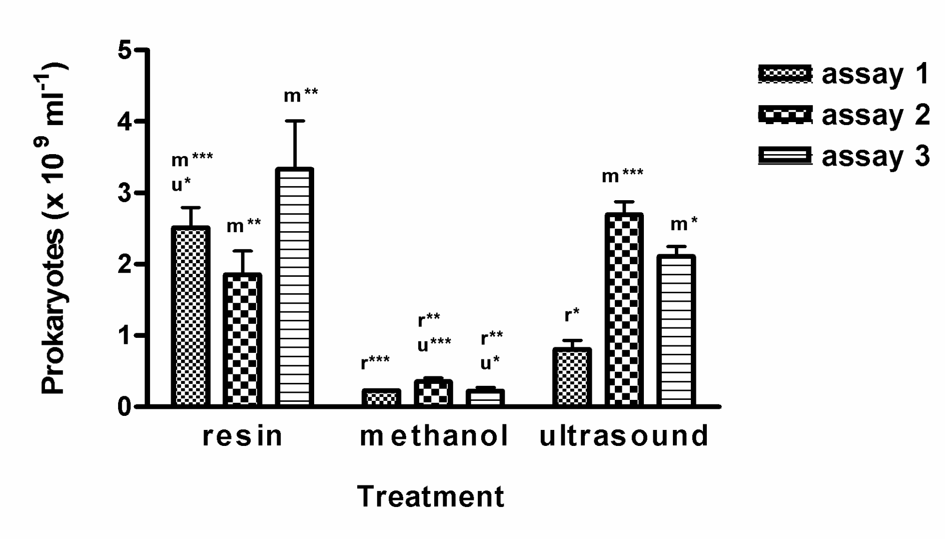

Fig. S2. Comparative test for the extraction of prokaryotes from muddy-sediment by dilution-ultrasound (our protocol) and by methanol (Lunau et al., 2005) or use of resin (Lucas et al., 1996). Reports of three assays performed in triplicate and analysed by microscopy (n=3, mean ± SE). Only significant differences intra-assays were reported: *: $p<0.05$; **: $p<0.01$; ***: $p<0.001$, with m= methanol extraction, u= use of ultrasound, r= use of resin. Our protocol was much more efficient (x 7.31 ± 3.79) compared with the methanol method; both were quite significantly related (r^2= 0.359; p=0.08; n=9) and could distinguish samples. The CV for the methanol extraction method was 32 ± 7% similar to the CV for our protocol (35 ± 8%), better than the CV for the resin recovery method (47 ± 7%). Resin method favored the extraction of prokaryotes (x 1.89 ± 1.44) for two sediments out of the three tested, without discriminating between the three sediments samples. To conclude, use of dilution-sonication method was considered as efficient for the sediment of the Marennes-Oléron Bay.
