# Supplementary material for: Viral Impact on Prokaryotic and Microalgal Activities in the Microphytobenthic Biofilm of an Intertidal Mudflat (French Atlantic Coast)
Source: Front Microbiol. 2015 Nov 10;6:1214. doi: 10.3389/fmicb.2015.01214 (PMC4639598; doi:10.3389/fmicb.2015.01214)
Supplement: Supplementary file 3 [file Image3.PDF]

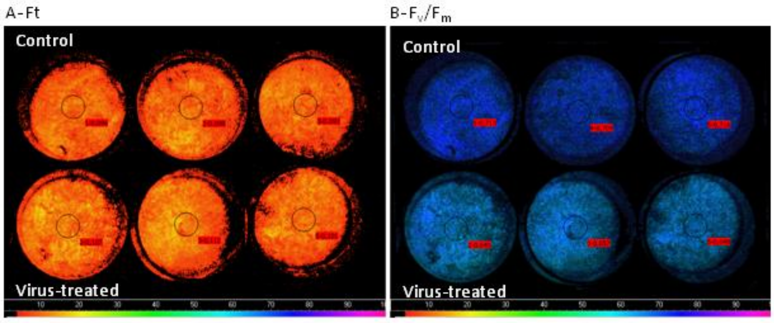

Fig. S3. False colour images of chlorophyll fluorescence and photosynthetic parameters obtained with a Maxi-Imaging-PAM fluorometer of untreated ('control') and a virus-treated (with Vb, five day infection) microphytobenthic biofilm in a 6-well microplate. Ft is the basal chlorophyll fluorescence which illustrates the microphytobenthic biomass, F_v/F_m is the maximum photosynthetic efficiency of photosystem II (PSII). Although Ft was similar in untreated and virus-treated sediments, the F_v/F_m was clearly lower in the virus-treated sediments, as illustrated by a light blue colouring compared to the strong blue colouring of 'control' wells.
