# Supplementary material for: Viral Impact on Prokaryotic and Microalgal Activities in the Microphytobenthic Biofilm of an Intertidal Mudflat (French Atlantic Coast)
Source: Front Microbiol. 2015 Nov 10;6:1214. doi: 10.3389/fmicb.2015.01214 (PMC4639598; doi:10.3389/fmicb.2015.01214)
Supplement: Supplementary file 4 [file Image4.PDF]

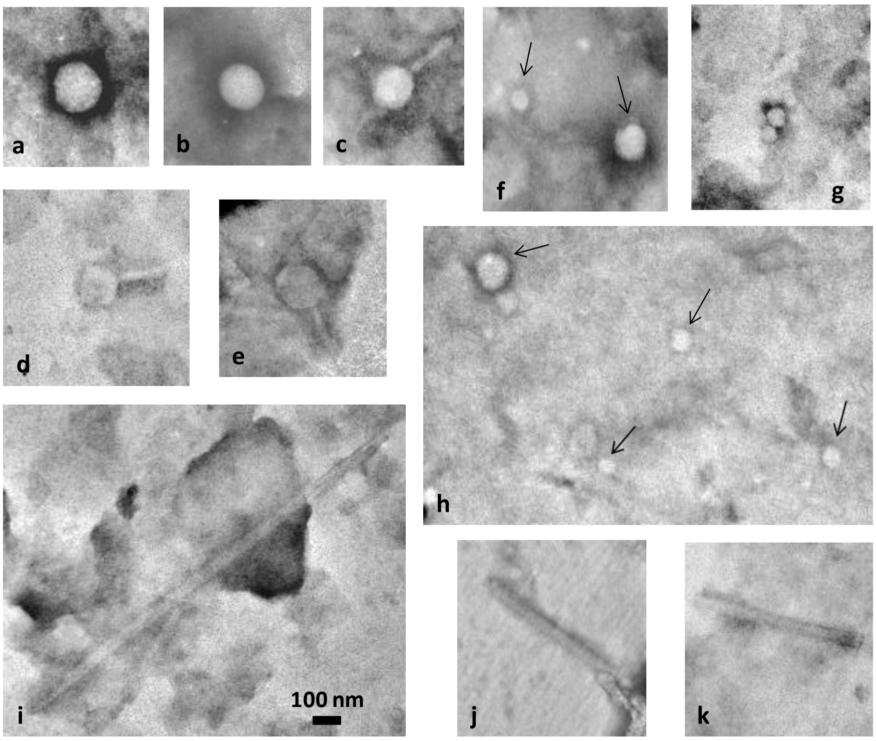

Fig. S4. Microphotographs of Virus Like Particles from top-surface sediment observed by TEM. Untailed icosaedrical particles (a,b,f,g,h), tailed particles (c,d,e) and filamentous shaped VLP (i,j,k). Arrows show distinguished VLP out of total particle like structure. Phosphotungstate staining, 50 000G, 120 Kvolt.
