# Supplementary material for: Viral Impact on Prokaryotic and Microalgal Activities in the Microphytobenthic Biofilm of an Intertidal Mudflat (French Atlantic Coast)
Source: Front Microbiol. 2015 Nov 10;6:1214. doi: 10.3389/fmicb.2015.01214 (PMC4639598; doi:10.3389/fmicb.2015.01214)
Supplement: Supplementary file 5 [file Image5.PDF]

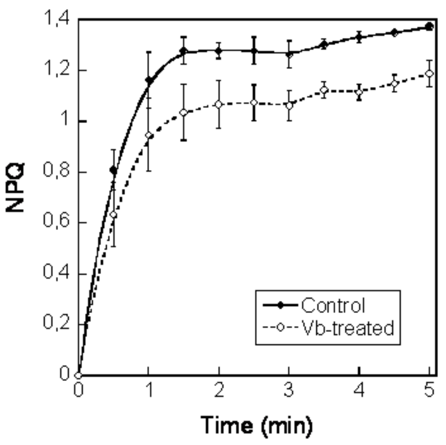

Fig. S5. Kinetics of non-photochemical fluorescence quenching (NPQ) development at a light intensity of 280 μmol·photons·m^{-2}·s^{-1} of controls (untreated) and viriobenthos (Vb)-treated microphytobenthic biofilm at five days post inoculation. Bioassays were performed in microwells with the top-surface sediment maintained, in May 2009.
